# Preoperative diagnosis and surgical treatment for giant retroperitoneal liposarcoma: A case report

**DOI:** 10.1016/j.ijscr.2019.04.003

**Published:** 2019-04-06

**Authors:** Anna Pisapia, Enrico Crolla, Rosario A. Saglioccolo, Alessandro Perrella, Carlo Molino

**Affiliations:** a Department of Oncological Surgery, A.O.R.N. “A. Cardarelli”, Naples, Italy; b VII Department of Infectious Disease and Immunology, Hospital “D. Cotugno”, Naples, Italy

**Keywords:** Liposarcoma, Retroperitoneum, Preoperative diagnosis, Compartmental surgery, Case report

## Abstract

**Introduction:**

The purpose of this article is to present the diagnostic and surgical approach for a giant retroperitoneal sarcoma and to highlight the difficulty of a precise preoperative diagnosis and the extention of surgical resection.

**Presentation of case:**

A 63-year-old female patient was admitted at our department with light diffuse abdominal pain, fever and gradual increase of abdominal girth. A CT scan showed a giant fatty tumor occupying left hemiabdomen and indirect findings of renal damage, probably sustained by ureter stretching and urine stasis. At surgical exploration, no cleavage plane was discovered between the mass and the surrounding organs. The severe pielonephritis and the apparent intraoperatively involvement of surrounding tumour structures lead to an aggressive surgery. An “en-bloc” resection of tumor mass, left colon, spleen, pancreatic tail, left annex, left kidney and adrenal gland was performed. Histology revealed a well differentiated liposarcoma with large areas of high grade of dedifferentiation.

**Discussion:**

Liposarcoma is an histologic subtype of soft tissue sarcoma and the most common type of sarcoma arising in retroperitoneum. It is difficult to make an accurate preoperative diagnosis through a percutaneous biopsy. Although it is required to obtain negative resection margins, literature shows that surgical radicality is not a primary endpoint if noble structures are strictly close. In some cases surgery must be more aggressive because of the apparent organ involvement or damage.

**Conclusion:**

This is a rare case of a giant liposarcoma involging many organs. Surgery should be tailored according to intraoperative findings and organ damage.

## Highlights

• Surgery should be tailored paying attention to noble structures.
• Damage or organ involvement requires an aggressive surgery.
• Sometimes close margins are necessary to preserve critical structures.

## 1 Introduction

Retroperitoneum is the primary site in about 15% of soft tissue sarcomas (STS) [1]. Liposarcomas represent the most common type of sarcoma arising in the retroperitoneum, about 45% [2]. Liposarcomas have four histologic subtypes: well-differentiated liposarcoma (WDLS), dedifferentiated liposarcoma (DDLS), myxoid/round cell liposarcoma, and pleomorphic liposarcoma.

Tumor behavior depends on the liposarcoma subtype. WDLS is locally aggressive but does not metastasize, whereas DDLS has the potential to metastasize (20–30% distant recurrence rate). DDLS also has a higher local recurrence rate than WDLS and six times the risk of death [3,4].

The rarity of retroperitoneal liposarcomas and the variety of histologic subtypes make it difficult to understand and treat this neoplasm [5]. Because of the large potential spaces of the retroperitoneum and local invasiveness of liposarcomas, these lesions are very large when diagnosed and often involve many adjacent organs and structures [5]. Surgery is the first step in resectable disease, however local recurrence rate can be >80% in DDLS [4].

Studies that have evaluated preoperative chemotherapy followed by surgery have reported inconsistent findings [6]. The results of the only randomized study that compared surgery alone versus preoperative chemotherapy followed by surgery in 134 evaluable patients with high-risk tumors (tumors ≥8 cm of any grade, grade 2/3 tumors <8 cm, grade 2/3 locally recurrent tumors, or tumors with inadequate surgery) did not show a major survival benefit for patients receiving chemotherapy [7].

We describe a case of a giant retroperitoneal liposarcoma and discuss about two still open question: the difficulty of a precise preoperative diagnosis and the extension of surgery.

This work is reported in line with the Surgical Case Report Guidelines (SCARE) criteria [8]

## 2 Presentation of case

**Photo 1:**
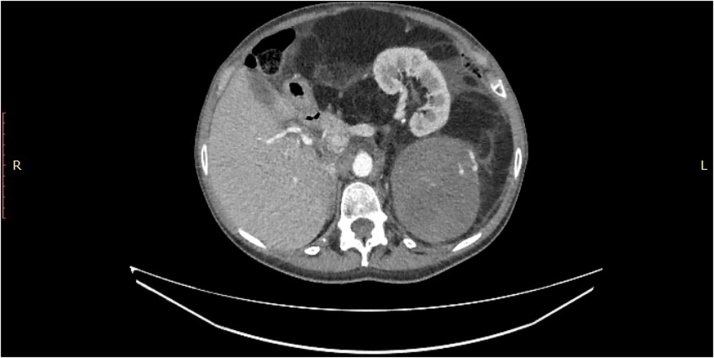
Left kidney dislocated in epigastrium by the retroperitoneal component of the mass.

**Photo 2:**
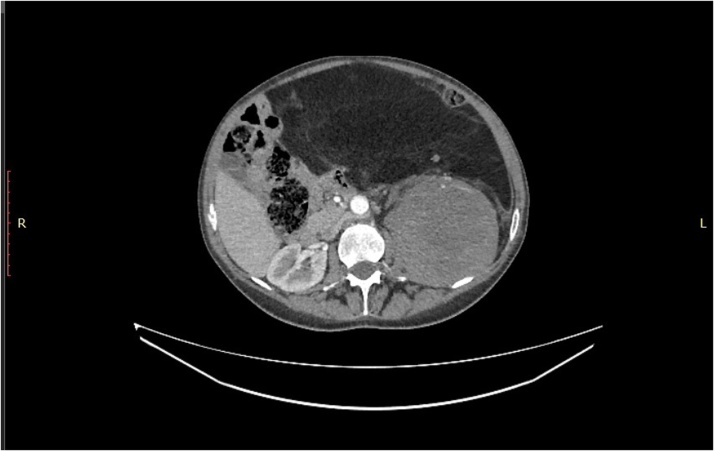
Left colon displaced against abdominal wall and most of the small bowel in right side of abdomen.

A 63-year-old female patient was admitted at our department. She reported a gradual increase of abdominal girth in the last months, light diffuse abdominal pain in the last two weeks and fever with chill in the last week. No other symptoms were reported. Physical examination on admission revealed a fixed abdominal mass with ill-defined margins occupying the entire abdomen. The patient was hemodynamically stable, HR 90 bpm, blood pressure 130/80 mmHg, oxygen saturation 95%, breath rate was 17, body temperature 37.8 °C and her bowel function was normal. BMI was 26.1. Laboratory tests revealed only a mild leukocytosis. Tumor markers were negative. Contrast enhanced CT showed a huge mass with a peritoneal component of 19 × 25 × 11 cm presenting a lipoma-like aspect with mixed density and pathological contrast enhancement, and a retroperitoneal uneven component of 10 × 14 × 5 cm. The mass displaced left kidney in epigastrium (Photo 1 ) and most of the bowel in right side of abdomen and pelvis (Photo 2 ). Renal collecting system was dilated and included dishomogeneous fluids and gas.

CT findings suggested a giant fatty tumor, including suspected dedifferentiated areas. Renal infection was probably sustained by ureter stretching and urine stasis. Surgery was decided.

**Photo 3:**
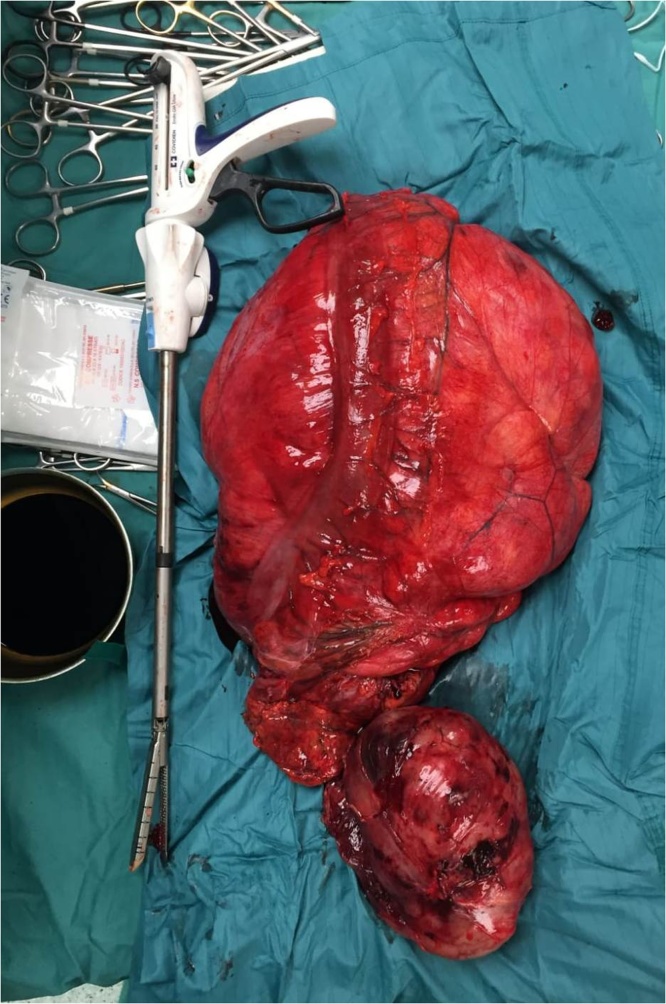
operative sample.

**Photo 4:**
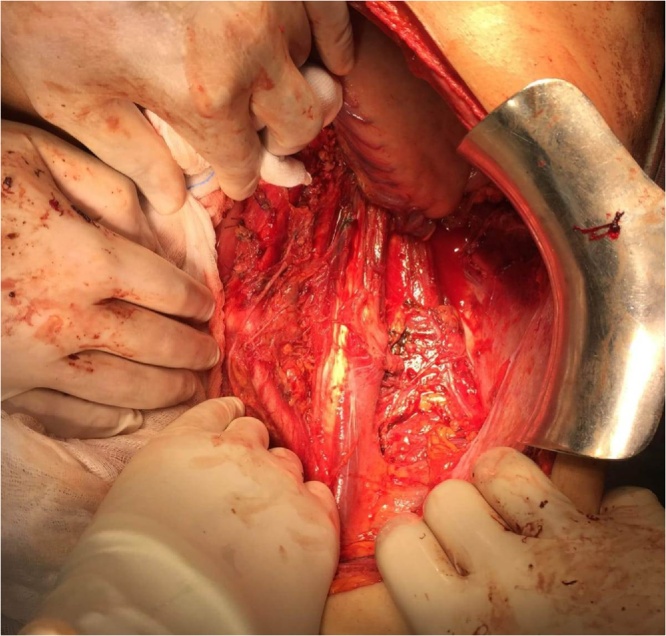
operating field after tumor resection with clips on vein renal stump.

A midline xyphoid to pubic incision was performed. Surgical exploration revealed a voluminous mass involving left colon, left kidney, spleen, tail of the pancreas and left uterine annex. No cleavage plane was discovered between the mass and the surrounding organs: so “en-bloc” resection of tumor mass, left colon, spleen, pancreatic tail, left annex, left kidney and adrenal gland was performed (Photo 3 ). Radiopaque clips were collocated on renal vein stump, as a marker, in order to guide a future radiotherapy (Photo 4 ). Colo-colic anastomosis and surgical drains placement completed the operation.

In the first postoperative days a decrease of urinary output occurred and a massive fluid reanimation was necessary. Postoperative anemia led to one RBC transfusion. The rest of recovery was uneventful: normal bowel functions restored in 2nd p.o. day and oral feeding restarted in 3rd p.o. day. Drains were removed in 7th p.o. day and the patient was discharged in 8th.

Histological examination showed a well differentiated liposarcoma, with lipoma-like and sclerosing aspects, with large areas of high grade of dedifferentiation (Ki67: 75%). Well differentiated component of the mass involved renal vein and left psoas margin (R1 resection).

## 3 Discussion

Fatty tumors of the retroperitoneum are rare and represent a real dilemma for the distinction between lipoma and well-differentiated liposarcoma. Computed tomography (CT) imaging features that suggest malignancy include large lesion size, presence of thick septa, presence of nodular and/or globular or non adipose mass-like areas, and decreased percentage of fat composition [9]. Moreover, within the fatty tumor can be present more histological types: an enhancing or centrally necrotic nodule may be indicative of a dedifferentiated component in well-differentiated liposarcoma [10].

Almost all liposarcomas occurring in the retroperitoneum are WDLS or DDLS [11].

Because of the difference of biological behavior of these two subtypes it is fundamental to determine the specific cellular population. Actually, liposarcoma present also an intra-tumoural variability and often we find various rates of the two components, describing them as DD/WD liposarcoma. A combination chemotherapy (doxorubicin and ifosfamide, the most common regime) versus single chemotherapy (single-agent doxorubicin) showed a response rate of 24% and a clinical benefit rate of 44%, higher than previously reported in DD liposarcoma; encouraging results also have been showed in recurrent or borderline-resectable patients who received combination chemotherapy as neoadjuvant therapy, either in DD liposarcoma or in the dedifferentiated cellular population rate of a giant liposarcoma [12].

A retrospective study on 256 patients underwent to surgical resection with pathologic results of WDLS and/or DDLS noted an unexpectedly low subtype-specific diagnostic accuracy of pre- operative image-guided percutaneous biopsy: 64% of DDLS patients and 15% of WDLS patients were incorrectly diagnosed with other types of tumors, and 53% of false-negative DDLS cases were diagnosed as WDLS [13]. Authors suggested two causes: technical sampling error, which may be prevent if images (CT or MRI scans) were reviewed at the time of biopsy, and the variable morphology of DDLS. Furthermore, the interface between well-differentiated and dedifferentiated areas is abrupt in most DDLS cases; occasionally, these tumors exhibit a mosaic pattern [14]. If a sample is obtained only from the well-differentiated component of the tumor, then the pathologic finding will be WDLS.

In this perspective, the poor accuracy of preoperative biopsy in distinguishing WDLS from DDLS, in the opinion of authors, could lead to avoid percutaneous sampling in the most part of cases, especially in symptomatic patients in which surgery is necessary.

Surgical resection with appropriate negative margins is the standard primary treatment for most patients with STS, although close margins may be necessary to preserve uninvolved critical neurovascular structures [6]. In addition to the difficulties to identify and consequently preserve retroperitoneal organs and structures, it must be said that high degrees of adipocyte differentiation in the tumor can pose difficulty at distinguishing it from retroperitoneal fat. Thus, the determination of a safe margin for resection becomes difficult [5].

According to guidelines of NCCN [6] radical excision/entire anatomic compartment resection is not routinely necessary. Extended resections involve visceral resection of colon, kidney, distal pancreas, diaphragm, spleen, and psoas.

Kirane and Crago [15] sustain that prophylactic compartmental resection has not been consistently shown to improve outcomes and is not universally accepted as standard of care: appropriate extent of “adequate” resection should be carefully considered based on grade, histology, and anatomy as it may not be justifiable in some disease types where local control is not the major determinant of survival.

If resections with microscopically positive or grossly positive margins are anticipated, surgical clips should be left in place to identify high-risk areas for recurrence, particularly for retroperitoneal or intra-abdominal sarcomas to help guide potential future radiotherapy [6].

Retroperitoneal liposarcoma tends to infiltrate adjacent tissues and structures with skip areas (satellites): for this reason it can be explained why it frequently recurs (from 6 to 24 months after initial surgery) despite complete surgical resection with negative margins [5,16].

Beyond these recent evidences there are some cases which need a “tailored” surgical strategy. In our case we report an anatomical condition in which an extent surgery is recommended of necessity. If we consider the severe pielonephritis due to the mechanical stretching on left ureter, nephrectomy was necessary. Nevertheless, other structures resected (descending colon, spleen, pancreatic tail, left annex) appeared intraoperatively involved in the fatty mass.

In the study by Singer et al. in 2003, the practice of dissecting under the renal capsule and reserving nephrectomy for instances of circumferential hilar involvement was not associated with a reduction in disease specific survival, 5 year OS reported at 92% [17].

Similarly, while aggressive vascular reconstruction is documented to be of low mortality at 4% with 5-year survival 66%, generally vessels can be freed of abutting tumor when not encased [17,18].

## 4 Conclusion

The surgical strategy adopted in this case, reveals authors’ opinion: an aggressive resection should not be performed “d’emblée”, whereas surgery should be tailored according to intraoperative findings: organ damage or organ engagement.

## Please state any conflicts of interest

No conflicts of interest.

## Please state any sources of funding for your research

No sources of funding.

## Ethical approval

As this work is a case report, ethical approval was not needed and our report was exempt from ethical approval at our institution.

## Consent

Written informed consent was obtained from the patient for publication of this case report and accompanying images. A copy of the written consent is available for review by the Editor-in-Chief of this journal upon request.

## Author contribution

Anna Pisapia: preparation of the manuscript, data interpretation, literature analysis.

Enrico Crolla: study planning, data interpretation.

Rosario Alessandro Saglioccolo: data collection, literature research.

Alessandro Perrella: data analysis.

Carlo Molino: study concept, data interpretation.

All authors read and approved final manuscript.

## Registration of research studies

As this is a case report and not a human studies, it is exempt from registering.

## Guarantor

Carlo Molino, MD.

## Provenance and peer review

Not commissioned, externally peer-reviewed.
